# Exploring the Appropriate Surgical Extent for Papillary Thyroid Carcinoma of the Isthmus: A Multicenter Retrospective Cohort Study

**DOI:** 10.3390/biomedicines14010013

**Published:** 2025-12-20

**Authors:** Yuhan Jiang, Yi Yang, Hanyun Tu, Tianyuchen Jiang, Anping Su

**Affiliations:** Division of Thyroid & Parathyroid Surgery, Department of General Surgery, West China Hospital Sichuan University, Chengdu 610041, China; 15508095003@163.com (Y.J.); yangyi00317@163.com (Y.Y.); angelatu666@163.com (H.T.); jiangtyc@163.com (T.J.)

**Keywords:** central lymph node metastasis, isthmus, papillary thyroid carcinoma, isthmus, surgical treatment, tumor recurrence

## Abstract

**Background:** Papillary thyroid carcinoma in the isthmus (PTCI) remains a subject of surgical debate due to its unique anatomical location and reportedly more aggressive behavior, including higher rates of lymph node metastasis, multifocality, extrathyroidal extension, and capsular invasion. There are currently no definitive guidelines regarding the optimal extent of surgery. **Objective:** This study aimed to compare the three surgical approaches—total thyroidectomy, lobectomy with isthmusectomy, and isthmusectomy/extended isthmusectomy—in patients with PTCI, focusing on postoperative complications, tumor recurrence, recovery, and identifying risk factors for tumor prognosis and lymph node metastasis. **Methods:** We retrospectively analyzed data from 215 patients treated surgically across four medical centers from 2016 to 2022, divided into three groups based on surgical extent. We compared baseline characteristics, operative time, intraoperative blood loss, length of hospital stay, postoperative complications, and central lymph node metastasis risk factors. Propensity Score Matching (PSM) was used to create more comparable groups, so as to verify the accuracy and stability of our research results. **Results:** No significant differences were observed among the three groups in rates of temporary or permanent recurrent laryngeal nerve injury, permanent hypoparathyroidism, or chyle leakage (all *p* > 0.05). However, transient hypoparathyroidism was more common in the total thyroidectomy group (*p* < 0.05), which also had longer operative time, greater intraoperative blood loss, and longer postoperative hospital stay (all *p* < 0.05) The PSM-adjusted analyses further confirmed these findings, except that the previously observed difference in postoperative drainage volume among the three groups was no longer significant (*p* = 0.791). The Kaplan–Meier curves showed a similar cumulative proportion of recurrence-free survivors in the three groups with no statistically significant difference observed (*p* = 0.804). Univariate and multivariate logistic regression analysis identified that gender (OR = 4.405, 95%CI: 4.104–4.729, *p* < 0.001), multifocality (OR = 2.498, 95%CI: 1.064–5.864, *p* = 0.035), tumor diameter (OR = 1.096, 95%CI: 1.047–1.147, *p* < 0.001), capsular invasion (OR = 2.666, 95%CI: 2.547–2.791, *p* < 0.001), and absolute eosinophil count (OR = 1.381, 95%CI: 1.125–1.695, *p* = 0.002) remained significant independent predictors of central lymph node metastasis in PTCI. A multivariable logistic regression model was developed to predict CLNM, achieving an AUC of 0.777. A probability threshold of 0.50 provided the best balance between sensitivity (77.6%) and specificity (65.5%) and was selected as the clinical cut-off for stratifying high- and low-risk patients. **Conclusions:** Conservative procedures like lobectomy with isthmusectomy or isthmusectomy/extended isthmusectomy may represent a feasible, function-preserving option in carefully selected low-risk PTCI patients, but further validation is required. In contrast, patients with high-risk features may benefit from central lymph node dissection. The predictive model may provide supportive information for personalized surgical planning.

## 1. Introduction

Thyroid cancer is the most common endocrine tumor. There are 821,000 new cases diagnosed worldwide as of 2022, ranking seventh in incidence among all malignant tumors [1]. PTC is the most common malignant tumor originating from thyroid follicular epithelial cells. PTC is mostly located in the thyroid lobes, while papillary thyroid carcinoma located in the isthmus is rare. The isthmus is located between the two lobes, with its posterior surface closely adjacent to the anterior wall of the trachea. It is relatively superficial in location, with the gland becoming thinner, narrower, and fewer in number in this area. Draw vertical lines from the outermost points on both sides of the trachea to the surface of the skin and designate the center point of the tumor as the intersection of its longest and shortest diameters. If the center point of the tumor is located between the two dotted lines, it is considered a isthmus thyroid tumor [2,3,4].

Previous studies comparing PTCI with lobe-derived PTC have suggested that PTCI exhibits more aggressive behavior, including higher rates of lymph node metastasis, multifocality, extrathyroidal extension, and capsular invasion [5,6,7]. This discrepancy has led surgeons to explore whether the surgical strategy for PTCI should differ from that for PTC of the lobes. On the one hand, some studies suggest that the adverse clinical pathological features of PTCI, such as gross extra-thyroidal extension, multifocality, and a tendency toward lymph node metastasis, make total thyroidectomy with bilateral central lymph node dissection an appropriate operative approach to reduce the risk of tumor recurrence and distant metastasis [8,9,10]. Wang et al. also conclude that for male patients with tumors larger than 0.6 cm and younger than 38 years, isthmusectomy may not be sufficient [6]. On the other hand, an increasing number of studies suggest that for PTCI, the smallest possible surgical field can reduce postoperative complications and improve postoperative quality of life while ensuring safety [3,11].

The controversy has persisted since the end of the 20th century, but the latest guidelines issued by authoritative bodies such as the ATA and the ETA have not made clear recommendations. However, the latest ATA guidelines published in 2025 have adjusted the optimal operative approach for differentiated thyroid carcinoma, recommending that lobectomy is sufficient for tumors smaller than 2 cm without gross extra-thyroidal extension [12]. This has also led us to consider whether a more conservative surgical scope could be selected for certain specific PTCIs than previously.

In this study, we attempted to compare the effects of different operative resection and lymph node dissection on postoperative recovery and long-term survival in PTCI, and to explore the risk factors for central lymph node metastasis, aiming to discuss the most appropriate operative plan and provide reference for the formulation of personalized therapy for PTCIs.

## 2. Materials and Methods

### 2.1. Patients

Data from 215 patients who received surgical therapy for PTCI at four tertiary-level comprehensive medical facilities between January 2016 and May 2022 were gathered retrospectively for this study. Patients between the ages of 18 and 70 who had had a preoperative thyroid ultrasound examination and who were believed to have PTCI based on FNAC or histology were included in our study. And also, none of the laboratory test results indicated absolute or uncontrolled relative contraindications, confirming the patients’ physical capacity to tolerate the surgical procedure and perioperative anesthesia safely. Patients were excluded if preoperative imaging revealed highly suspicious malignant nodules within the thyroid lobes without definitive malignant involvement of the isthmus, or if they underwent lateral neck lymph node dissection. We analyzed the age, gender, BMI, Hashimoto’s thyroiditis, nodular goiter, multifocality, thyroid-related serum marker levels, and blood inflammation levels of all included cases. Ethical approval for this study was obtained from the Institutional Review Boards of all four participating medical centers. All data collected were obtained with informed consent from the patients. All operations were performed by three or more surgeons with at least 15 years of experience in thyroid and parathyroid surgery. To ensure consistency in pathological diagnostic criteria, all postoperative specimens and slides from the four participating centers were centrally reviewed at the Department of Pathology, West China Hospital of Sichuan University. The reviews were conducted by two expert pathologists, each with more than 15 years of experience in thyroid tumor pathology, and the consensus review served as the final basis for analysis. The choice of operative approach for total thyroidectomy, thyroidectomy with isthmusectomy, isthmusectomy, and extended isthmusectomy is primarily determined by factors such as tumor location, size, capsular invasion, multifocality, and patient preference. Postoperative iodine-131 radioactive iodine is applied to selected high-risk patients. For low-risk patients with no residual disease after surgery, the therapeutic goal is to maintain serum TSH levels at the lower end of the normal range or within the low-normal range. For intermediate-to-high-risk patients, individualized TSH-suppressive therapy should be implemented. Tumor recurrence is monitored through tests such as thyroid globulin and thyroid ultrasound.

### 2.2. Definitions

The 8th edition of the TNM system of the AJCC was used for tumor staging. The tumor center was defined as the intersection point of its longest and shortest diameters. A tumor was classified as an isthmic tumor if its center fell between the medial borders of the trachea. Tumors whose centers were located within the medial tracheal borders but extended beyond the tracheal margin were also categorized as isthmic tumors. Lesions confirmed as papillary thyroid carcinoma on postoperative pathology were classified as PTCI. Lobectomy with isthmusectomy was defined as resection of the thyroid isthmus together with the ipsilateral thyroid lobe, with or without resection of the medial one-third of the contralateral lobe adjacent to the isthmus. Isthmusectomy/extended isthmusectomy was defined as resection of the thyroid isthmus alone, with or without removal of the medial one-third of both thyroid lobes adjacent to the isthmus. Tumor recurrence was defined as the recurrence of the thyroid carcinoma in the operative area or distant metastasis. All cases of recurrence were confirmed by pathology or imaging. Postoperative complications included transient and permanent hoarseness and hypoparathyroidism, as well as chyle leakage. Postoperative measurements of serum PTH and calcium levels were obtained for all patients on day one. Temporary hypoparathyroidism was defined as a postoperative PTH level below 15 pg/mL or a serum calcium concentration below 2.1 mmol/L, or the presence of symptoms such as muscle pain, facial or limb numbness, tetany, or hyperactive tendon reflexes. If these conditions persisted for more than six months, the condition was classified as permanent hypoparathyroidism. Permanent hoarseness was characterized by persistent postoperative voice hoarseness lasting ≥6 months, where recurrent laryngeal nerve paralysis was confirmed via laryngoscopy. The primary outcome measures were tumor-free survival and postoperative complications, while secondary outcome measures included surgical duration, intraoperative blood loss, and postoperative hospital stay.

### 2.3. Statistical Analysis

SPSS 26.0 and Python 3.10 software were used to analyze the data. Continuous variables with a normal distribution, verified using the Kolmogorov–Smirnov test, were expressed as means ± standard deviations and compared using one-way analysis of variance. Continuous variables that did not follow a normal distribution were expressed as medians with interquartile ranges and compared using the Kruskal–Wallis test. For categorical variables expressed as absolute values and percentages, the chi-square test and Fisher’s exact test (two-tailed) were used to analyze heterogeneity. Post hoc pairwise comparisons were performed using Dunn’s test, with Bonferroni correction applied when necessary. Tumor recurrence outcomes were analyzed using the Kaplan–Meier curve. Univariate and multivariate logistic regression analysis was used to analyze the risk factors for central lymph node metastasis. PSM was performed using Python software. Propensity scores were estimated through logistic regression based on the following covariates: age, sex, BMI, maximum tumor diameter, multifocality, capsular invasion, extrathyroidal extension, recurrent laryngeal nerve invasion, and tracheal invasion. A 1:1 nearest-neighbor matching without replacement was applied, using a caliper width of 0.1. *p*-values less than 0.05 were considered statistically significant.

## 3. Results

### 3.1. Patient Characteristics

A total of 215 PTCI patients who received initial treatment between 2016 and 2022 and met the criteria were included. Among them, 157 (73%) were female and 58 (27%) were male. The average age of the patients was 42.99 (±11.90) years old, and the maximum diameter of the tumors ranged from 9 (5 to 12) mm. In total, 4 cases (1.9%) had hyperthyroidism, 6 cases (2.8%) had hypothyroidism, 43 cases (20.0%) had Hashimoto’s thyroiditis, and 103 cases (47.9%) were diagnosed with nodular goiter. Multifocality was identified in 39 cases (18.1%). Based on the extent of lymph node dissection, patients were divided into three groups: 21 (9.8%) received dissection limited to the pretracheal and prelaryngeal areas, 47 (21.9%) underwent unilateral central compartment dissection, and 147 (68.3%) received bilateral central compartment dissection. Postoperatively, 54 patients (25.1%) deemed at high risk of recurrence received I-131 radioactive iodine therapy. The median follow-up time for the entire cohort was 29 months. Detailed information is shown in Table 1.

### 3.2. Surgical Groups

Based on the different operation ranges, patients were categorized into three groups: those who underwent total thyroidectomy (*n* = 121), unilateral lobectomy with isthmusectomy (*n* = 50), or isthmusectomy/extended isthmusectomy alone (*n* = 44) (Table 2). No statistically significant differences were observed among the three groups regarding gender, age, or underlying thyroid diseases (*p* > 0.05). There was a statistically significant discrepancy in BMI levels between patients in the total thyroidectomy group (total thyroidectomy: 24.83 ± 4.23; unilateral lobectomy with isthmusectomy: 23.83 ± 3.77; isthmusectomy/extended isthmusectomy: 22.77 ± 2.90, *p* = 0.024). A statistically significant difference in preoperative TSH levels was observed among the three groups (total thyroidectomy: 2.57 (1.78–4.19); unilateral lobectomy with isthmusectomy: 1.97 (1.36–2.88); isthmusectomy/extended isthmusectomy: 1.78 (1.1–2.24) *p* < 0.001). Furthermore, the PLR levels also differed significantly among at least two groups (total thyroidectomy: 100 (74.58–134.85); unilateral lobectomy with isthmusectomy: 106.56 (88.15–153.40); isthmusectomy/extended isthmusectomy: 123.53 (99.70–178.39); *p* = 0.028). The distribution of the largest tumor diameter among the three groups showed a statistically significant discrepancy (*p* < 0.001). Pairwise comparisons showed that the tumor diameter was significantly larger in the total thyroidectomy group compared to both the unilateral lobectomy with isthmusectomy group (*p* < 0.001) and the isthmusectomy/extended isthmusectomy group (*p* < 0.001). No significant difference was observed between the latter two groups (*p* = 0.241). This pattern may reflect surgical preference bias, as patients presenting with larger tumor diameters on preoperative imaging (e.g., ultrasound or CT) are more likely to be recommended total thyroidectomy.

The chi-square test indicated significant differences in tumor multifocality among the three groups (*p* = 0.020). Further subgroup analyses with pairwise comparisons showed that the unilateral lobectomy with isthmusectomy group had a significantly higher incidence of multifocality compared with the isthmusectomy/extended isthmusectomy group (*p* = 0.022). In contrast, no significant differences were observed between the total thyroidectomy group and the other two groups (all *p* > 0.05). It should be noted that the multifocality described in this study refers to pathological multifocality, which includes both multiple intraisthmic lesions and microcarcinomas located in the ipsilateral or contralateral thyroid lobe in addition to the primary isthmus tumor. These small lobar foci were generally not detected on preoperative ultrasound but were identified only upon postoperative histopathological examination. A significant difference in extrathyroidal extension was also identified among the three groups (*p* = 0.008). Post hoc analysis indicated a higher incidence in the total thyroidectomy group relative to the unilateral lobectomy with isthmusectomy group (*p* = 0.031) and the isthmusectomy/extended isthmusectomy group (*p* = 0.006), whereas no significant difference was observed between the latter two groups (*p* = 0.563). This discrepancy may be attributed to selection bias during surgical decision-making, as surgeons are more inclined to opt for total thyroidectomy when multifocality or extrathyroidal extension is present. The results of pairwise comparisons between groups are presented in Table 3.

### 3.3. Perioperative Outcomes and Complications

We specifically compared three surgical approaches in terms of operative time, blood loss, and postoperative recovery. The median operative time was 140 (118–166) minutes in the total thyroidectomy group, 110 (79.5–138) minutes in the unilateral lobectomy with isthmusectomy group, and 91 (77–144.25) minutes in the isthmusectomy/extended isthmusectomy group, with a statistically significant difference among the three groups (*p* < 0.001). Post hoc pairwise comparisons revealed that the total thyroidectomy group had a significantly longer operative time compared to both the lobectomy with isthmusectomy group (*p* < 0.001) and the isthmusectomy/extended isthmusectomy group (*p* < 0.001). However, no statistically significant difference was observed between the latter two groups (*p* = 0.723). A significant intergroup difference was also noted in intraoperative blood loss (*p* < 0.001). No statistically significant difference was detected between the two lesser resection groups (*p* = 0.399). Likewise, statistically significant variations were observed in both length of postoperative hospitalization (*p* < 0.001) and postoperative drainage volume (*p* = 0.028) among the surgical approaches. Patients undergoing total thyroidectomy had a longer postoperative stay compared to those in the other two groups (*p* < 0.05), and also exhibited significantly greater drainage volume than the isthmusectomy/extended isthmusectomy group (*p* = 0.015).

We conducted a statistical analysis of common postoperative complications associated with thyroid surgery and found no statistically significant differences among the three groups in terms of temporary or permanent recurrent laryngeal nerve injury, permanent hypoparathyroidism, or chyle leakage (all *p* > 0.05). However, a significant difference was observed in the incidence of transient hypoparathyroidism (*p* < 0.001). Further pairwise comparisons revealed that the total thyroidectomy group had a significantly higher rate of transient hypoparathyroidism compared to the other two groups (*p* < 0.05), while no significant difference was detected between the unilateral lobectomy with isthmusectomy group and the isthmusectomy/extended isthmusectomy group (*p* = 0.618).

### 3.4. Perioperative Outcomes and Complications After PSM

Given the baseline differences among the three surgical groups in tumor diameter, ETE, and multifocality, PSM was performed to reduce confounding. Propensity scores were calculated using age, sex, BMI, maximum tumor diameter, multifocality, capsular invasion, extrathyroidal extension, recurrent laryngeal nerve invasion, and tracheal invasion as covariates. After 1:1 matching, the three groups were well balanced, with no statistically significant differences in baseline characteristics (all *p* > 0.05), indicating good matching quality.

A total of 130 patients were included in the analysis. As shown in Table 4, significant differences persisted among the three groups in operative time (total thyroidectomy: 131.5 [110.75–149.50] min; unilateral lobectomy with isthmusectomy: 95.00 [76.75–130.25] min; isthmusectomy/extended isthmusectomy: 94.00 [66.25–136.00] min; *p* < 0.001), intraoperative blood loss (30.00 [12.50–50.00] mL vs. 10.00 [10.00–20.00] mL vs. 10.00 [5.00–20.00] mL; *p* < 0.001), and postoperative length of stay (4.00 [3.00–5.00] days vs. 2.00 [2.00–4.00] days vs. 3.00 [2.00–5.00] days; *p* < 0.001). In contrast, postoperative drainage volume showed no significant differences among the groups (*p* = 0.791). Pairwise comparisons demonstrated a stepwise increase in operative time and length of stay with expanding surgical extent (all *p* < 0.05). Intraoperative blood loss was significantly higher in the total thyroidectomy group compared with the unilateral lobectomy with isthmusectomy group (*p* = 0.028) and the isthmusectomy/extended isthmusectomy group (*p* < 0.001), while no difference was observed between the latter two groups (*p* = 0.056).

Regarding postoperative complications, the only variable that remained significantly different among the three groups was transient hypoparathyroidism (*p* = 0.002). Pairwise analysis further showed that transient hypoparathyroidism was more common in the total thyroidectomy group compared with the unilateral lobectomy with isthmusectomy group (*p* = 0.009) and the isthmusectomy/extended isthmusectomy group (*p* < 0.001), whereas no difference was found between the two more conservative approaches (*p* = 0.162) (Table 5).

### 3.5. Tumor Recurrence and Survival

The median follow-up time for the entire cohort was 29 months (range: 15–49.5). The median follow-up durations for the three groups were 42 months (22–66) for the total thyroidectomy group, 23 months (12–31.75) for the unilateral lobectomy with isthmusectomy group, and 15.5 months (12–27) for the isthmusectomy/extended isthmusectomy group, respectively. Tumor recurrence was observed in only three patients (2.5%), all of whom belonged to the total thyroidectomy group. No tumor-related deaths occurred during the follow-up period. Consequently, no statistically significant difference in recurrence rates was observed among the three groups (*p* = 0.307). After PSM, only one patient (1.8%) in the total thyroidectomy group experienced tumor recurrence, and no statistically significant differences in tumor recurrence were observed among the three groups (*p* = 0.503) (Table 4).

**Figure 1:**
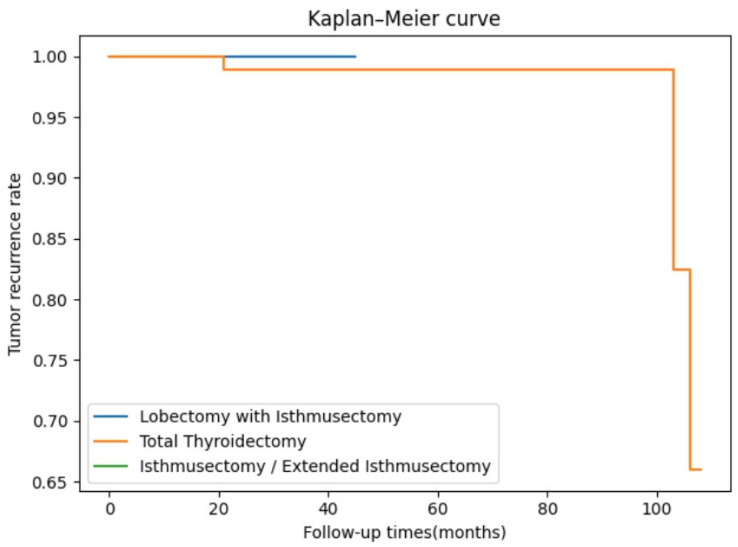
The Kaplan–Meier survival curve of the cumulative proportion of recurrence-free survivors in the three groups.

However, it is noteworthy that the total thyroidectomy group had a significantly longer follow-up period compared to the other two groups (*p* < 0.001). Therefore, the Kaplan–Meier survival curve provides us with the cumulative proportion of recurrence-free survivors in the three groups. In this study, the recurrence-free survival curves based on the different surgical approaches showed no statistically significant difference (*p* = 0.804) (Figure 1).

### 3.6. Potential Risk Factors for Central Lymph Node Metastasis

We further categorized the 215 PTC patients into groups based on their lymph node metastasis status and compared their baseline characteristics, aiming to preliminarily explore the clinicopathological factors associated with central lymph node metastasis in PTC. As shown in Table 6, patients with central lymph node metastasis had a significantly higher proportion of males (*p* < 0.001), tumor multifocality (*p* = 0.011), and capsular invasion (*p* = 0.031) compared to those without central lymph node metastasis. We found that patients with central lymph node metastasis generally had larger tumor diameter (*p*< 0.001), higher preoperative SII (*p* = 0.04), NMPLR (*p* < 0.001), and eosinophil absolute count (*p* = 0.009) levels.

On the basis of the above, we included these factors in a univariate logistic regression analysis for exploratory purposes (Table 7). To reduce verification bias related to differing extents of lymph node dissection, only 136 patients who underwent bilateral central lymph node dissection were included in the analysis. In the multivariable logistic regression, “different center” was specified as a clustering variable, and cluster-robust standard errors were used to account for within-center correlations. The results indicated that gender (OR = 3.856, 95% CI: 1.602–9.281, *p* = 0.003), capsular invasion (OR = 3.627, 95% CI: 1.495–8.796, *p* = 0.004), largest tumor diameter (OR = 1.081, 95% CI: 1.018–1.148, *p* = 0.011), and absolute eosinophil count (OR = 1.611, 95% CI: 1.086–2.390, *p* = 0.018) were significantly associated with central lymph node metastasis. However, tumor multifocality, SII, and NMPLR lost significance in the univariate logistic regression model (all *p* > 0.05).

These significant variables were further incorporated into a multivariate logistic regression analysis, which confirmed that gender (OR = 4.405, 95%CI: 4.104–4.729, *p* < 0.001), multifocality (OR = 2.498, 95%CI: 1.064–5.864, *p* = 0.035), tumor diameter (OR = 1.096, 95%CI: 1.047–1.147, *p* < 0.001), capsular invasion (OR = 2.666, 95%CI: 2.547–2.791, *p* < 0.001), and absolute eosinophil count (OR = 1.381, 95%CI: 1.125–1.695, *p* = 0.002) remained significant independent predictors of central lymph node metastasis in PTCI. (Table 7).

**Figure 2:**
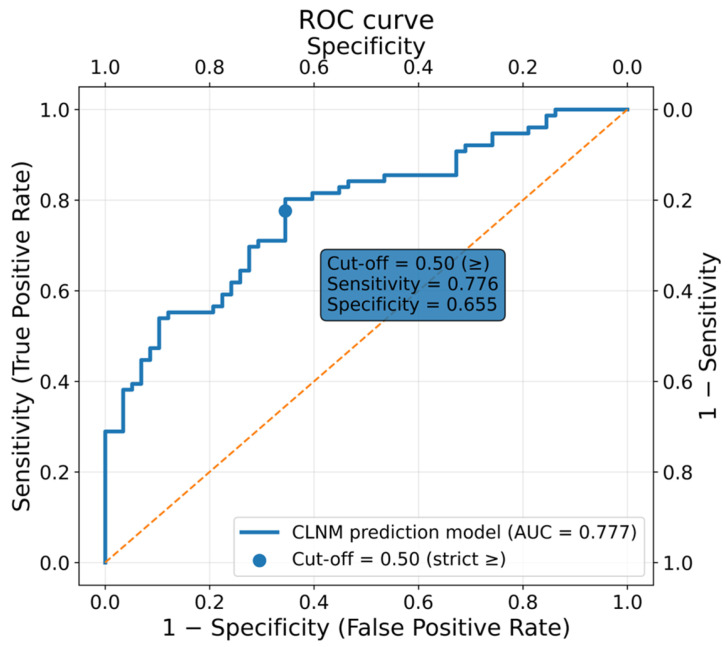
ROC curve of the predictive model for central lymph node metastasis in PTCI. (The yellow dashed diagonal line represents the reference line indicating no discriminative ability (AUC = 0.5)). Abbreviations: AUC, Area under the curve.

Using a multivariable logistic regression model, we established a predictive model for central lymph node metastasis (CLNM), which demonstrated good discriminatory performance, with an AUC of 0.777 (Figure 2). To enhance clinical interpretability, we further evaluated the model’s sensitivity and specificity across a range of predicted probability thresholds. A threshold of 0.50 provided the most balanced performance, yielding a sensitivity of 77.6%, a specificity of 65.5%, and a Youden index of 0.431, representing the optimal trade-off among the candidate cut-off values. Therefore, we selected 0.50 as the key clinical decision threshold for stratifying patients into high- and low-risk groups for CLNM (Table 8).

## 4. Discussion

### 4.1. Overview of Findings

At present, no consensus guideline specifically addresses the optimal surgical extent for PTCI, and controversies mainly concern how much thyroid and lymphatic tissue should be removed. According to the ATA guideline and existing evidence, the choice of surgery should consider tumor size, focality, and nodal status [12]. Isthmusectomy/extended isthmusectomy—removal of the isthmus alone or together with the medial one-third of both lobes—offers the advantage of avoiding exposure of the tracheoesophageal groove, thereby reducing the risk of recurrent laryngeal nerve and parathyroid injury. In contrast, lobectomy with isthmusectomy and total thyroidectomy provide wider exposure and more thorough clearance but carry higher surgical morbidity. Given the unique anterior location of the isthmus and its proximity to the trachea, PTCI demonstrates distinct anatomical behavior. With improved ultrasound detection, its reported incidence has risen to 2.2–17.4% in recent studies [13,14].

### 4.2. Surgical Strategy Debate

Previous studies have shown that although isthmus nodules account for only 6% of all thyroid nodules, they carry the highest malignancy risk [15] and are more frequently associated with extrathyroidal extension, lymph node metastasis, capsular invasion, and multifocality compared with lesions in the thyroid lobes [16,17,18]. Owing to the thin isthmic tissue [8] and its close anatomical relationship with the trachea and strap muscles, PTCI has traditionally been regarded as requiring more aggressive surgery.

Based on these features, several studies have recommended total thyroidectomy for PTCI, reporting higher rates of capsular invasion and multifocality [8], increased central lymph node metastasis [19], and lower recurrence after total thyroidectomy [10]. Other researchers, however, have suggested that less extensive procedures may also provide adequate control in selected patients. Skilbeck et al. reported favorable outcomes after isthmusectomy [20], and Sugenoya et al. observed no recurrences over more than 20 years after isthmusectomy in selected cases [21]. A meta-analysis also found no significant differences in recurrence or complications between total and less-than-total thyroidectomy [22]. These findings collectively indicate that the optimal surgical extent for PTCI remains controversial.

In our multicenter cohort, the rates of multifocality, capsular invasion, and extrathyroidal extension were comparable to those reported in prior studies [8,14], supporting the notion that PTCI is often intermediate-to-high-risk. After PSM, perioperative outcomes, complication profiles, and short-term recurrence rates were largely comparable among unilateral lobectomy with isthmusectomy, isthmusectomy/extended isthmusectomy, and total thyroidectomy, with the exception of a higher incidence of transient hypoparathyroidism following total thyroidectomy, consistent with previous observations [4]. Although all tumor recurrences occurred in the total thyroidectomy group, this group had more aggressive baseline features and markedly longer follow-up, limiting direct comparison. Kaplan–Meier analysis showed no significant difference among the three procedures.

Three patients experienced disease recurrence during follow-up. The first patient was found to have supraclavicular lymph node metastasis at 18 months postoperatively. The patient subsequently underwent left selective neck dissection. At 36 months after the initial surgery, a left supraclavicular lymph node metastasis was again detected and treated with microwave ablation. The second patient was diagnosed with suprasternal lymph node metastasis 44 months after surgery and underwent a second lymph node dissection. The third patient was detected with bilateral lung metastases merely three months post operation and received radioactive iodine therapy at a dose of 100 mCi. All three patients showed favorable responses to subsequent treatment and remained alive without disease-related mortality at the last follow-up. Given the small number of recurrence events and follow-up imbalance, long-term oncologic equivalence cannot yet be determined.

Clinically, our findings support a more individualized, risk-adapted approach rather than routine total thyroidectomy strategy for all PTCIs. Total thyroidectomy remains appropriate for high-risk presentations such as gross ETE, extensive multifocality, or the anticipated need for postoperative radioactive iodine. In contrast, unilateral lobectomy with isthmusectomy and isthmusectomy/extended isthmusectomy demonstrated favorable functional outcomes and lower rates of hypoparathyroidism [23], suggesting that these procedures may be suitable alternatives in selected patients. Therefore, increasing evidence—including our own results—indicates that in carefully selected low-risk PTCI patients (for example, ≤1 cm, unifocal disease, no clinical lymph node involvement, and no extrathyroidal extension), isthmusectomy or extended isthmusectomy may provide adequate tumor control while minimizing surgical morbidity. These function-preserving approaches may represent a valid surgical option for highly selected PTCI cases. Importantly, any inference regarding long-term oncologic safety should be interpreted cautiously and requires confirmation through larger prospective studies with extended follow-up.

### 4.3. Lymph Node Metastasis Risk

The latest ATA guidelines no longer recommend routine CLND for most cN0 papillary thyroid carcinomas, particularly small, non-invasive tumors. Prophylactic CLND is reserved only for selected T3–T4 tumors or when the results would directly influence postoperative management [12]. However, the lymphatic spread pattern of PTCI remains controversial. Lee et al. reported that although PTCI had a lower rate of lateral lymph node metastasis, the risk of CLNM was similar to that of lobar PTC [8]. In contrast, another study found a significantly higher CLNM rate in PTCI compared with lobe-based tumors, with no significant difference in lateral metastasis [23]. These discrepancies may largely stem from selection bias and inconsistent CLND extent across studies.

The 2025 ATA guidelines emphasize the Delphian node as a frequent site of lymphatic spread in PTCI [12]. Anatomically, the isthmus drains primarily into the pretracheal and Delphian nodes [7]. This unique drainage pattern explains the higher frequency of prelaryngeal nodal involvement in PTCI and its known association with increased risks of central and even lateral metastasis [24]. Moreover, Delphian node metastasis has been repeatedly linked to poorer prognosis and decreased DSS and OS in head and neck malignancies [25]. Accordingly, Iyer et al. recommend intraoperative frozen section evaluation of the prelaryngeal lymph node, and if positive, a more comprehensive central lymph node assessment should be considered [24].

In our study, CLNM was identified in approximately half of patients, partly reflecting our inclusive definition of central compartment involvement. Male, larger tumor size, multifocality, capsular invasion, and elevated inflammatory markers were more prevalent in the N1a group, consistent with prior studies [8,26,27]. Univariate and multivariate analyses further confirmed that sex, multifocality, capsular invasion, tumor diameter, and absolute eosinophil count were independent predictors of CLNM, whereas the significance of SII diminished after adjustment for confounders. The emergence of multifocality as a significant factor only after adjustment likely reflects its correlation with other aggressive features.

We further developed a predictive model for CLNM based on the identified independent factors. The model achieved an AUC of 0.777, with a clinically meaningful threshold of 0.50 providing a balanced sensitivity and specificity. While statistically valid, this performance indicates that the model should be regarded as an exploratory and adjunctive tool, rather than a definitive risk stratification instrument. Its potential value lies in assisting preoperative risk assessment and supporting individualized decision-making regarding CLND, particularly in balancing the risk of nodal disease against procedure-related morbidity.

### 4.4. Study Strengths and Limitations

We acknowledge that our study has several limitations. Firstly, despite being larger than many previous PTCI series, the overall sample size remains limited, restricting statistical power, particularly for recurrence analysis. Secondly, as a retrospective study, it is susceptible to selection bias. Additionally, we were unable to adjust for postoperative RAI therapy and the degree of TSH suppression in the tumor recurrence analysis, both of which are known to influence long-term prognosis in PTC. And the median follow-up duration of 29 months is relatively short, and late recurrences may not yet be captured. Furthermore, our study specifically excluded large tumors (≥4 cm), as these are rare in the isthmus due to anatomical constraints, and their management typically mandates total thyroidectomy based on established guidelines. The more aggressive nature of larger tumors, often associated with extrathyroidal extension and metastasis, could confound the assessment of surgical extent. Therefore, our conclusions are primarily applicable to the more commonly encountered smaller PTCIs, which was the focus of this investigation. Future prospective, multicenter studies with longer follow-up are required to validate these findings and further define the role of conservative surgical strategies in PTCI.

## 5. Conclusions

Our findings suggest that unilateral lobectomy with isthmusectomy and isthmusectomy/extended isthmusectomy appears to be a function-preserving alternative to total thyroidectomy in carefully selected low-risk patients with PTCI, particularly those with small, unifocal tumors and no evidence of lymph node metastasis. These more conservative procedures are associated with lower complication rates and better postoperative recovery, and no significant difference in short-term recurrence was observed between different surgical strategies. However, given the rarity of recurrence events and the unequal duration of follow-up among groups, the long-term oncologic equivalence remains to be confirmed. By contrast, in male patients with multifocality, capsular invasion, larger tumor diameter, or elevated preoperative absolute eosinophil counts, performing a central compartment lymph node dissection appears to be a safer oncologic strategy, and at minimum pretracheal and prelaryngeal node dissection should be undertaken, with intraoperative frozen section of the Delphian node to guide the decision for prophylactic CLND when metastasis is detected. In addition, the CLNM prediction model, with an AUC of 0.777 and a clinically relevant cut-off of 0.50, yielding balanced sensitivity (77.6%) and specificity (65.5%), demonstrated moderate discrimination and may be used as an exploratory adjunct for preoperative risk assessment and individualized surgical decision-making in PTCI, pending further external validation.

## Figures and Tables

**Table 1:** Demographic and clinicopathologic characteristics of all patients.

Variables	Value
Gender, *n* (%)	
Male	58 (27%)
Female	157 (73%)
Age, mean ± SD (years)	42.99 ± 11.90
BMI, mean ± SD (kg/m^2^)	23.91 ± 3.81
Comorbidity, *n* (%)	
Hypertension	30 (14%)
Diabetes Mellitus	10 (4.7%)
Coronary Artery Disease	5 (2.3%)
Hyperthyroidism	4 (1.9%)
Hypothyroidism	6 (2.8%)
Hashimoto’s Thyroiditis	43 (20%)
Nodular Goiter	103 (47.9%)
Maximum Tumors Diameter (mm)	9 (5–12)
Tumor Characteristics, *n* (%)	
Multifocality	39 (18.1%)/176 (81.9%)
Capsular Invasion	160 (74.4%)/55 (25.6%)
Extrathyroidal Extension	46 (21.4%)/169 (78.6%)
Recurrent Laryngeal Nerve Invasion	3 (1.4%)/212 (98.6%)
Tracheal Invasion	5 (2.3%)/210 (97.7%)
Surgical Extent, *n* (%)	
Total Thyroidectomy	121 (56.3%)
Lobectomy with Isthmusectomy	50 (23.3%)
Isthmusectomy/Extended Isthmusectomy	44 (20.4%)
Lymph Node Dissection, *n* (%)	
Pretracheal and Prelaryngeal Dissection	21 (9.8%)
Unilateral Central Neck Dissection	47 (21.9%)
Bilateral Central Neck Dissection	147 (68.3%)
Radioactive Iodine Ablation, *n* (%)	54 (25.1%)/161 (74.9%)
Follow-up (months)	29 (15–49.5)

Abbreviations: SD, standard deviation; BMI, body mass index.

**Table 2:** Clinical and pathological characteristics of patients by type of surgical procedure (pre PSM).

Variables	Total Thyroidectomy (*n*= 121)	Lobectomy with Isthmusectomy (*n* = 50)	Isthmusectomy/Extended Isthmusectomy (*n* = 44)	*p*-Value
Gender, *n* (%)				0.131
Male	34 (28.1%)	17 (34%)	7 (15.9%)	-
Female	87 (71.9%)	33 (66%)	37 (84.1%)	-
Age, mean ± SD (year)	43.65 ± 12.63	43.14 ± 11.98	40.98 ± 9.53	0.442
BMI, mean ± SD (kg/m^2^)	24.83 ± 4.23	23.83 ± 3.77	22.77 ± 2.90	0.024
Maximum Tumor Diameter of the Tumors (mm)	10 (7–15)	6 (5–9.5)	5.5 (4–8.25)	<0.001
Tumor Characteristics, *n* (%)				
Multifocality	24 (19.8%)	13 (26%)	2 (4.5%)	0.020
Capsular Invasion	93 (76.9%)	39 (78%)	28 (63.6%)	0.182
Extrathyroidal Extension	35 (28.9%)	7 (14%)	4 (9.1%)	0.008
Recurrent Laryngeal Nerve Invasion	2 (1.7%)	0 (0)	1 (2.3%)	0.603
Tracheal Invasion	4 (3.3%)	0 (0%)	1 (2.3%)	0.427
BRAF Mutation	25 (20.7%)	11 (22%)	8 (18.2%)	0.898
Medical history				
Hashimoto’s Thyroiditis	27 (22.5%)	5 (10%)	11 (25%)	0.119
Nodular Goiter	62 (51.2%)	25 (50%)	16 (36.4%)	0.226
Preoperative Labs				
TSH (mIU/L)	2.57 (1.78–4.19)	1.97 (1.36–2.88)	1.78 (1.1–2.24)	<0.001
FT3 (pmol/L)	4.69 (4.38–5.41)	4.64 (4.37–4.99)	4.69 (4.35–5.03)	0.719
FT4 (pmol/L)	16.8 (15.43–18.07)	15.7 (14.15–17.35)	15.8 (15.00–17.30)	0.260
Tg (ng/mL)	16.2 (5.19–28.5)	18.6 (12.4–28.55)	12.1 (8.53–19.6)	0.316
TgAb (IU/mL)	15.9 (11.7–299)	15.7 (13.95–27.85)	15.4 (13.75–78.4)	0.712
SII	309.95 (206.09–399.03)	327.62 (289.67–442.42)	436.33 (286.29–560.75)	0.209
NLR	1.69 (1.40–2.11)	1.83 (1.40–2.20)	1.91 (1.35–2.29)	0.267
MLR	0.22 (0.18–0.26)	0.21 (0.17–0.25)	0.21 (0.17–0.28)	0.595
PLR	100 (74.58–134.85)	106.56 (88.15–153.40)	123.53 (99.70–178.39)	0.028
NMPLR	110.17 (68.14–155.62)	125.9 (80.12–177.23)	190.91 (81.22–261.61)	0.640
Surgical Parameters				
Operative Time (min)	140 (118–166)	110 (79.5–138)	91 (77–144.25)	<0.001
Intraoperative Blood Loss (mL)	30 (20–50)	10 (10–20)	10 (10–20)	<0.001
Parathyroid Autotransplantation (*n*)	1 (1–2)	1 (0–1)	1 (0–1)	<0.001
Postoperative Drainage (mL)	82 (50–130)	75 (47.5–106)	72.5 (50–90.5)	0.028
Postoperative Hospital Stay (days)	4 (3–5)	2 (2–5)	3 (2–5)	<0.001
Postoperative Complications, *n* (%)				
Hoarseness				-
Transient	6 (5%)	2 (4%)	0 (0%)	0.328
Permanent	1 (0.8%)	0 (0%)	0 (0%)	0.677
Hypoparathyroidism				-
Transient	63 (52.1%)	7 (14%)	4 (9.1%)	<0.001
Permanent	3 (2.5%)	0 (0%)	0 (0%)	0.307
Chyle Leak	1 (0.8%)	0 (0%)	1 (2.3%)	0.511
Tumor Recurrence, *n* (%)	3 (2.55%)	0 (0%)	0 (0%)	0.307
Follow-up Time (months)	42 (22–66)	23 (12–31.75)	15.5 (12–27)	<0.001

Abbreviations: TSH, Thyroid Stimulating Hormone; FT3, Free Triiodothyronine; FT4, Free Thyroxine; Tg, thyroid globulin; TgAb, Thy-Roglobulin Antibody; SII, Systemic Immune-Inflammation Index; NLR, Neutrophil-to-Lymphocyte Ratio; MLR, Monocyte-to-Lymphocyte Ratio; PLR, Platelet-to-Lymphocyte Ratio; NMPLR, Neutrophil-Monocyte-Platelet-to-Lymphocyte Ratio.

**Table 3:** Post hoc pairwise comparisons between groups (*p*-values) for significant variables (pre PSM).

Variables	A vs. B	A vs. C	B vs. C
Multifocality	0.342	0.074	0.022
Operative Time	<0.001	<0.001	0.723
Intraoperative Blood loss	<0.001	<0.001	0.399
Extrathyroidal Extension	0.031	0.006	0.563
Largest Tumor Diameter	<0.001	<0.001	0.241
Parathyroid Autotransplantation	<0.001	<0.001	0.522
Postoperative Hospital Stay	<0.001	0.002	0.085
Transient Hypoparathyroidism	<0.001	<0.001	0.618
Postoperative Drainage	0.107	0.015	0.529

Abbreviations: A: total thyroidectomy; B: lobectomy with isthmusectomy; C: isthmusectomy/extended isthmusectomy.

**Table 4:** Clinical and pathological characteristics of patients by type of surgical procedure (after PSM).

	Total Thyroidectomy (*n* = 55)	Lobectomy with Isthmusectomy (*n* = 41)	Isthmusectomy/ Extended Isthmusectomy (*n* = 34)	*p*-Value
Gender, *n* (%)				0.183
Male	11 (20%)	13 (31.7%)	5 (14.7%)	
Female	44 (80%)	28 (68.3%)	29 (85.3%)	
Age, mean ± SD (years)	42.69 ± 12.05	42.22 ± 11.99	40.50 ± 9.72	0.673
BMI, mean ± SD (kg/m^2^)	23.59 ± 3.49	23.68 ± 4.02	23.07 ± 2.99	0.740
Maximum Tumor Diameter (mm)	7.00 (5.00–10.00)	7.00 (5.00–10.00)	6.00 (5.00–9.00)	0.616
Tumor Characteristics, *n* (%)				
Multifocality	10 (18.2%)	8 (19.5%)	2 (5.9%)	0.199
Capsular Invasion	37 (68.5%)	31 (75.6%)	23 (69.7%)	0.736
Extrathyroidal Extension	8 (14.5%)	7 (17.1%)	4 (11.8%)	0.811
Recurrent Laryngeal Nerve Invasion	1 (1.8%)	0 (0.0%)	1 (2.9%)	0.574
Tracheal Invasion	0 (0.0%)	0 (0.0%)	1 (2.9%)	0.241
Surgical Parameters				
Operative Time (min)	131.50 (110.75–149.50)	95.00 (76.75–130.25)	94.00 (66.25–136.00)	<0.001
Intraoperative Blood Loss (mL)	30.00 (12.50–50.00)	10.00 (10.00–20.00)	10.00 (5.00–20.00)	<0.001
Postoperative Drainage (mL)	70.00 (50.00–101.00)	77.50 (55.00–101.50)	70.00 (50.00–90.00)	0.791
Postoperative Hospital Stay (days)	4.00 (3.00–5.00)	2.00 (2.00–4.00)	3.00 (2.00–5.00)	<0.001
Postoperative Complications, *n* (%)				
Hoarseness				
Transient	4 (7.3%)	2 (4.9%)	0 (0.0%)	0.282
Permanent	1 (1.8%)	0 (0.0%)	0 (0.0%)	0.503
Hypoparathyroidism				
Transient	23 (41.8%)	7 (17.1%)	4 (11.8%)	0.002
Permanent	2 (3.6%)	0 (0.0%)	0 (0.0%)	0.250
Chyle Leak	0 (0.0%)	0 (0.0%)	1 (2.9%)	0.241
Tumor Recurrence, *n* (%)	1 (1.8%)	0 (0.0%)	0 (0.0%)	0.503
Follow-up Time (months)	44.73 ± 28.47	21.54 ± 10.68	17.19 ± 12.14	<0.001

**Table 5:** Post hoc pairwise comparisons between groups (*p*-values) for significant variables (after PSM).

Variables	A vs. B	A vs. C	B vs. C
Operative Time	0.017	<0.001	0.043
Intraoperative Blood Loss	0.028	<0.001	0.056
Postoperative Hospital Stay	0.021	<0.001	0.037
Transient Hypoparathyroidism	0.009	<0.001	0.162

Abbreviations: A: total thyroidectomy; B: lobectomy with isthmusectomy; C: isthmusectomy/extended isthmusectomy.

**Table 6:** Basic characteristics of positive and negative central lymph node metastasis patients.

Variables	N0 (*n* = 106)	N1a (*n* = 109)	*p*-Value
Gender, *n* (%)			<0.001
Male	17 (16%)	41 (37.6%)	-
Female	89 (84%)	68 (62.4%)	-
Age, mean ± SD (year)	44.5 ± 11.31	41.51 ± 12.33	0.066
BMI, mean ± SD (kg/m^2^)	23.51 ± 3.71	24.40 ± 3.89	0.151
Medical history, *n* (%)			
Nodular Goiter	57 (53.8%)	46 (42.2%)	0.089
Hashimoto’s Thyroiditis	26 (24.8%)	17 (15.6%)	0.094
Tumor Characteristics, *n* (%)			
Multifocality	12 (11.3%)	27 (24.8%)	0.011
Capsular Invasion	72 (67.9%)	88 (80.7%)	0.031
Extrathyroidal Extension	19 (17.9%)	27 (24.8%)	0.221
Recurrent Laryngeal Nerve Invasion	1 (0.9%)	2 (1.8%)	0.577
Tracheal Invasion	1 (0.9%)	4 (3.7%)	0.185
Tumor Location, *n* (%)			0.432
Center	43 (40.6%)	50 (45.9%)	-
Lateral	63 (59.4%)	59 (54.1%)	-
Maximum Tumor Diameter (mm)	6 (5–10)	10 (6–15)	<0.001
Preoperative Labs			
TSH (mIU/L)	2.02 (1.47–3.04)	1.94 (1.3–2.97)	0.710
FT3 (pmol/L)	4.56 (4.31–5.04)	4.92 (4.54–5.21)	0.067
FT4 (pmol/L)	15.92 ± 2.63	15.04 ± 5.07	0.108
Tg (ng/mL)	15.35 (8.66–26.03)	16.9 (7.00–28.70)	0.514
TgAb (IU/mL)	16.15 (12.90–81.15)	15.6 (13.95–70.2)	0.827
SII	339.98 (237.85–458.58)	367.25 (281.10–486.14)	0.040
NLR	1.83 (1.33–2.15)	1.72 (1.43–2.22)	0.421
MLR	0.21 ± 0.07	0.22 ± 0.08	0.378
PLR	107.02 (87.24–150.62)	109.62 (86.61–141.56)	0.553
NMPLR	120.71 (71.13–168.19)	149.97 (87.06–245.85)	<0.001
Calcium Level (pg/mL)	2.30 (2.24–2.35)	2.29 (2.26–2.38)	0.340
Magnesium Level (mmol/L)	0.91 ± 0.07	0.90 ± 0.07	0.307
Phosphorus Level (mmol/L)	1.12 ± 0.24	1.10 ± 0.18	0.579
Absolute Eosinophil Count (10^9^/L)	0.08 (0.05–0.14)	0.11 (0.06–0.18)	0.009
Absolute Basophil Count (10^9^/L)	0.02 (0.02–0.04)	0.03 (0.02–0.04)	0.152
CEA (ng/mL)	1.19 (0.78–1.97)	1.31 (0.92–1.72)	0.578

Abbreviations: N0, PTCI without regional lymph node metastasis; N1a, PTCI with central lymph node metastasis; TSH, Thyroid Stimulating Hormone; FT3, Free Triiodothyronine; FT4, Free Thyroxine; Tg, thyroid globulin; TgAb, Thy-Roglobulin Antibody; SII, Systemic Immune-Inflammation Index; NLR, Neutrophil-to-Lymphocyte Ratio; MLR, Monocyte-to-Lymphocyte Ratio; PLR, Platelet-to-Lymphocyte Ratio; NMPLR, Neutrophil-Monocyte-Platelet-to-Lymphocyte Ratio; CEA, Carcinoembryonic antigen.

**Table 7:** Univariate and multivariate logistic regression analysis of risk factors for central lymph node metastasis in PTCI.

Variables	Univariate			Multivariate		
	OR	95% CI	*p*-Value	OR	95% CI	*p*-Value
Gender	3.856	1.602–9.281	0.003	4.405	4.104–4.729	<0.001
Multifocality	1.943	0.741–5.092	0.177	2.498	1.064–5.864	0.035
Capsular Invasion	3.627	1.495–8.796	0.004	2.666	2.547–2.791	<0.001
Largest Tumor Diameter	1.081	1.018–1.148	0.011	1.096	1.047–1.147	<0.001
SII	1.002	1.000–1.004	0.058	1.003	1.000–1.006	0.049
NMPLR	1.003	1.000–1.007	0.053	0.998	0.994–1.003	0.410
Absolute Eosinophil Count	1.611	1.086–2.390	0.018	1.381	1.125–1.695	0.002

Abbreviations: OR, Odds ratio; CI, Confidence interval; SII, Systemic Immune-Inflammation Index; NMPLR, Neutrophil-Monocyte-Platelet-to-Lymphocyte Ratio.

**Table 8:** Sensitivity, Specificity, and Youden Index of the CLNM prediction model at different probability cut-off values.

Cut-Off	Sensitivity	Specificity	Youden Index
0.077	1.000	0.034	0.034
0.200	0.987	0.155	0.142
0.300	0.921	0.276	0.197
0.400	0.855	0.466	0.321
0.500	0.776	0.655	0.431

## Data Availability

The data presented in this study are available on request from the corresponding author due to they involve patient privacy.

## Abbreviations

The following abbreviations are used in this manuscript:

ATA	American Thyroid Association
AJCC	American Joint Committee on Cancer
AUC	Area Under the ROC Curve
BMI	Body Mass Index
CLNM	Central Lymph Node Metastasis
CLND	Central Lymph Node Dissection
DSS	Disease-Specific Survival
ETA	European Thyroid Association
ETE	Extrathyroidal Extension
FNAC	Fine Needle Aspiration Cytology
FT3	Free Triiodothyronine
FT4	Free Thyroxine
MLR	Monocyte-to-Lymphocyte Ratio
NLR	Neutrophil-to-Lymphocyte Ratio
NMPLR	Neutrophil-Monocyte-Platelet-to-Lymphocyte Ratio–
OS	Overall Survival
PTCI	Papillary Thyroid Carcinoma in the Isthmus
PSM	Propensity Score Matching
PLR	Platelet-to-Lymphocyte Ratio
PTH	Parathyroid Hormone
RAI	Radioactive Iodine
SII	Systemic Immune Inflammation Index
TSH-Suppressive	Thyroid-Stimulating Hormone-Suppressive
TgAb	Thyroglobulin Antibody
